# Inter-doctor variations in the assessment of functional incapacities by insurance physicians

**DOI:** 10.1186/1471-2458-11-864

**Published:** 2011-11-14

**Authors:** Antonius JM Schellart, Henny Mulders, Romy Steenbeek, Johannes R Anema, Herman Kroneman, Jan Besseling

**Affiliations:** 1 VU University Medical Center, Department of Public and Occupational Health/EMGO Institute for Health, Amsterdam, the Netherlands; 2 UWV, Dutch Employee Insurance Authority Amsterdam, the Netherlands; 3 TNO Work and Health, Hoofddorp, the Netherlands; 4 Research Center for Insurance Medicine AMC-UMCG-UWV-VUmc, Amsterdam, the Netherlands

## Abstract

**Background:**

The aim of this study was to determine the - largely unexplored - extent of systematic variation in the work disability assessment by Dutch insurance physicians (IPs) of employees on long-term sick leave, and to ascertain whether this variation was associated with the individual characteristics and opinions of IPs.

**Methods:**

In March 2008 we conducted a survey among IPs on the basis of the 'Attitude - Social norm - self-Efficacy' (ASE) model. We used the ensuing data to form latent variables for the ASE constructs. We then linked the background variables and the measured constructs for IPs (n = 199) working at regional offices (n = 27) to the work disability assessments of clients (n = 83,755) and their characteristics. These assessments were carried out between July 2003 and April 2008. We performed multilevel regression analysis on three important assessment outcomes: No Sustainable Capacity or Restrictions for Working Hours (binominal), Functional Incapacity Score (scale 0-6) and Maximum Work Disability Class (binominal). We calculated Intra Class Correlations (ICCs) at IP level and office level and explained variances (R^2^) for the three outcomes. A higher ICC reflects stronger systematic variation.

**Results:**

The ICCs at IP level were approximately 6% for No Sustainable Capacity or Restrictions for Working Hours and Maximum Work Disability Class and 12% for Functional Incapacity Score. Background IP variables and the measured ASE constructs for physicians contributed very little to the variation - at most 1%. The ICCs at office level ranged from 0% to around 1%. The R^2 ^was 11% for No Sustainable Capacity or Restrictions for Working Hours, 19% for Functional Incapacity Score and 37% for Maximum Work Disability Class.

**Conclusion:**

Our study uncovered small to moderate systematic variations in the outcome of disability assessments in the Netherlands. However, the individual characteristics and opinions of insurance physicians have very little impact on these variations. Our findings provided no indications of other reasons for these variations. They may be related to different work routines or to different views on the workload of a 'normal' employee. If so, they could be reduced by well-developed and comprehensively implemented guidelines. Therefore, further research is needed.

## Background

### Introduction

Taking decisions in complex situations by professionals (e.g. in law, finance) implies that similar decisions may lead to variation in outcome between professionals. This is a universal given and physicians are no exception. The problem of so called 'inter-doctor variation' goes back a long way and was even highlighted by Montaigne (1595), the famous French essayist [1,2]. Various, more recent studies show that physicians can arrive at different diagnostic interpretations and prescribe different treatments for patients with the same clinical symptoms and anamnesis [3]. Eisenberg [4,5] has collated various reasons for inter-doctor variation, drawn from different studies [3]. These include practice style, personal characteristics, the influence of colleagues, and the degree of uncertainty inherent in the practice of medicine and the way doctors deal with it. Eisenberg's study suggests that variation increases in proportion to the level of uncertainty. Long [6], on the other hand, stresses 'patient agency constraints', a collective term for organizational and environmental constraints, and argues that once all the variables that constitute these constraints have been identified, the remainder represents 'innate variance', or differences in practice style. Furthermore, Nilsen et al. [7] identified in a qualitative study under 48 general practitioners (GPs) in Norway the following factors that might influence decision-making on sick-listing patients: the patients' ability to present their story to evoke sympathy, the GPs' prior knowledge of the patient, the GPs' own experience as a patient, and the GPs' tendency to avoid conflicts. The authors [7] conclude that issuing sickness certification was mainly patient-driven, and the decisions vary according to GPs' attitudes, beliefs, and personalities.

### The Dutch social security system for work disability assessment

In the Netherlands, insurance physicians work for private insurance companies or in the social insurance sector. In this study we investigate inter-doctor variation in disability assessments of long-term sick-listed employees by insurance physicians working in the social insurance sector. The actual assessment process, carried out by an insurance physician and a labour expert, is described in more detail in other studies [[8-10], Broersen JPJ, Mulders HPG, Schellart AJM, Van der Beek AJ: The Identification of Job Opportunities for Severely Disabled Sick-listed Employees, submitted].

In the Netherlands, if you are partially or fully incapable of working after two years of illness, you may be eligible to receive a benefit under the Work and Income Act (WIA). The WIA succeeded the Disability Insurance Act (WAO) in January 2006. The Adapted Re-assessment Act (HERBO) was introduced in August 2004 for the reassessment of WAO benefits clients on the basis of new, stricter criteria that put the emphasis on the client's residual functional capacities. The WAO and WIA differ in the time of assessment: the WAO provides for assessments after one year of sick-leave, whereas the WIA provides for assessments after two years. Insurance physicians have to judge a client's 'claim' to compensation for loss of work capacity. They base their decision on the information in the dossier, on information from the client and other parties, and on additional medical examinations. The insurance physician carries out this assessment within a statutory framework, supplemented with regulations and guidelines and - more recently - diagnosis-specific protocols for measuring the functional capacity of employees on long-term sick leave.

The findings about the residual (post morbid) functional abilities of a client serve as input for the labour expert (LE) in determining with the Claim Assessment and Monitoring System, CAMS [11], to which extent the residual functional abilities of the client match with the work demands of jobs, and to which extent the client may earn income with these jobs theoretically. When this theoretical income of matching jobs is less than the normal standard income of the client, the LE computes the loss of income, i.e. the theoretical income as a percentage of the normal standard income. This percentage determines the degree of work disability: lightly disabled (< 35% disability, partially disabled (35-80% disability), or fully disabled (80-100%). These degrees are assigned temporarily, i.e. full or partial recovery is still possible. Clients who are assessed by the insurance physician as fully disabled on medical grounds with no possibility of recovery (i.e. no sustainable capacity left), are declared fully disabled (80-100% disability) until the time that they are eligible for work pension.

### Disability assessments by insurance physicians

In order to assess a clients' work limitations the insurance physician starts with the functional limitations experienced by the client. These are tested for plausibility and internal and external consistency on the basis of the medical history and the actual ability of the client to perform. The insurance physician bases his assessment on an interview and possibly a physical examination. He can obtain additional information by ordering additional tests or by contacting the GP, specialist or other healthcare provider, or the occupational physician (OP) who assessed the first two years of disability. For clients with severe pathology and impairments and with no chance on recovery, the insurance physician may assess full disability on medical ground: no sustainable capacity left. For all other clients, the client's capacity for work is determined by reference to an instrument known as Functional Ability List (FAL). As an instrument the FAL comes within the statutory framework of disability assessments in the Netherlands. On this list the physician enters the client's scores for limitations and abilities. The FAL consists of 106 items, categorised into six sections: personal functioning, social functioning, adjusting to the physical environment, dynamic movements, static posture, and working hours. In about 75% of the cases the insurance physician assesses the functional capacity of the client by applying the Functional Abilities List (FAL) [8,11]. The remaining 25% of cases comprises two types of client: a) those who, in the professional opinion of the insurance physician, can resume their original job; and b) those who are very severely disabled, possibly bedridden [8]. As an instrument, the FAL forms part of the statutory framework of disability assessments in the Netherlands.

In the Netherlands disability assessment instruments have been the subject of a number of studies [12-15]. These studies, however, have taken place in a laboratorial rather than a 'real life' context. Recently, Spanjer [16] published a study in which thirty-one pairs of insurance physicians independently assessed a real-life client with low back pain (62 patients). However, to the best of our knowledge, no large-scale studies were conducted on inter-doctor variation in disability assessments. This study is of international interest because assessments of work disability take place in many countries [17-19] and inter-doctor variation in these assessments may play a role as well.

### Research questions

In 2004 the Dutch Council for Health Care Research (CHR), a government body, drew attention to the need to research inter-doctor variation in disability assessments performed by insurance physicians in the Netherlands [20]. The central theme of our study is therefore the judgments of Dutch social insurance physicians in compensation claims for loss of income due to diminished work capacity. We were interested primarily in insurance physicians and the role of their office (i.e. their work environment) as a source of variation, but, at the same time, expected client characteristics to influence the outcome of the assessments.

In a previous study [9] we developed a conceptual framework for the variables that constitute important sources of inter-doctor variation. We then validated the measured constructs in a structural model [10], which describes the extent to which attitude, social norms and self-efficacy influence the self-reported intentions and behavioural characteristics of insurance physicians, while taking account of knowledge, barriers and background variables. This study is a continuation on the two previous studies and sought to find answers to four questions:

A) What is the variation in relevant outcome measures of disability assessments at insurance physician level (IP level) and at regional office level?

B) How strong is the association between the client background variables and the outcome measures?

C) Which background variables, opinions and considerations of the insurance physicians themselves are associated with the outcome measures of disability assessments?

D) How strong is the association of these outcome measures with IP level and office level, respectively?

## Methods

### Study design and procedure

We collected information about social insurance physicians and their clients from two sources. The first source is a one-off survey in March 2008 to measure the background variables, opinions, and considerations of insurance physicians. The questions in the survey about opinions and considerations of insurance physicians were based on the 'Attitude - Social norm - self-Efficacy' model (ASE model) [21], an extension of the Theory of Planned Behaviour [22], which incorporates Bandura's concept of self-Efficacy [23] and the concepts of Knowledge and Barriers [24]. At the end of this survey we asked the respondents for permission to link their answers to the assessments results of their clients. More detailed descriptions can be found in publications elsewhere [9,10]. The second source is the Claim Assessment and Monitoring System (CAMS) of the Dutch Employee Insurance Authority, a database with assessment results of all clients. In May 2008 we extracted data on disability assessments at client level from this database [25] of. The linkage procedure - to link the survey data of the insurance physician respondents to the assessments results of their clients - was carried out in August 2008 by a trusted third party (an external agency) under the supervision of a public notary. All data concerning clients, physicians and their regional offices were anonymized before they were handed over to the researchers. As this study was a secondary analysis of anonymized data, approval by a Medical Ethical Commission was not necessary under Dutch law.

### Study population

The target group consisted of insurance physicians actively employed by the Employee Insurance Authority in May 2008 who performed assessments of long-term sick-listed employees in 2007 or in preceding years, within three comparable statutory regimes for long-term work disability: WAO; HERBO and WIA. Physicians who were not working for the Dutch Employee Insurance Authority in March 2008 and physicians who performed assessments only for other regimes (i.e. ZW: short term sick leave of workers without an employer, and WAJONG: invalidity pension for young disabled persons) were excluded, because these regimes cover other populations and use other assessment criteria to determine disability. Our estimate was that the target group consisted of 450 insurance physicians. Two hundred and thirty-one insurance physicians (approximately 51% of the target group) responded to the survey, of whom 200 gave informed consent to link their survey answers with the client database. For further details on the study population of insurance physicians we refer to Steenbeek et al [9].

We created a total client group from the three above-mentioned regimes so that the assessments could be analysed together. The inclusion criteria were: a) the client data could be linked with the insurance physician data, and b) the assessment period for the three regimes, i.e. from July 2003 to March 2005 for the WAO regime, from November 2004 to December 2006 for the HERBO regime, and from January 2006 to April 2008 for the WIA regime. All the clients were aged between 15 and 65 at the time of the assessment and suffered from one or more of the diseases or disorders in Version 10 of the International Code of Diseases [26]. The survey data were linked for 199 insurance physicians in 27 regional offices and 91,149 clients (linkage failed for one physician who gave informed consent). Next, we further selected data by applying two criteria: 1) we selected physicians with 10 or more clients per office to be sure of sufficient reliability in the multivariate analysis, and 2) we assigned each physician to the office where he/she had the most clients, meaning that clients attending the office(s) where the physician had fewer clients were excluded from the analysis, this to avoid cross-classified multilevel modelling. The first criterion resulted in the removal of three physicians and reduced the number of clients to 91,139. The second resulted in 83,755 clients assessed by 196 insurance physicians in 27 regional offices.

### Outcome measures for disability assessments

The following data at client level on the results of disability assessments by insurance physicians were extracted from the CAMS-database a) no sustainable functional capacities (i.e. the client has zero working capacities left now and will not recover in the future); b) restrictions in working hours; c) functional capacities per FAL item; and d) work disability class. From the extracted data we constructed three outcome measures (Table 1 and Table 2). 1) 'No sustainable functional capacities, extended (NSCE)': a combination of 'no sustainable functional capacities' and 'restrictions to 30 working hours or less'. The measure 'no sustainable functional capacities' - which leads to the maximum work disability class 80-100% per definition - was combined with 'restrictions in working hours' (to a maximum of 30 per week) which, in practice, also led to no jobs. This because the system that labour experts worked with did not contain many part-time jobs at that time (2003-2007). When the labour expert had to specify jobs for clients who could not work for more than 30 hours a week, the CAMS-system often came up with no jobs which then resulted in an assessment of these clients in the maximum work disability class 80-100%. We therefore merged the two outcomes into one outcome measure. 2) 'Functional incapacity score (FIS)'. Broersen et al. [[8], Broersen JPJ, Mulders HPG, Schellart AJM, Van der Beek AJ: The Identification of Job Opportunities for Severely Disabled Sick-listed Employees, submitted] investigated the factor structure of the FAL-items and distinguished four incapacity scales: mental incapacities, physical incapacities, incapacity to function autonomously and incapacities of the function of the hands. Because of a poor reliability of the fourth scale in our study (Cronbach's alpha < 0.5), we distinguished three incapacity scales: mental incapacities, physical incapacities (now including incapacities of the hands) and incapacity to function autonomously. The three scales had moderate reliability (Cronbach's alphas: 0.69, 0.72 and 0.75 respectively). Because these three scales were highly skewed, we transformed them into three incapacity variables with three categories each, ordered from zero to two (see Table 2). The outcome measure 'functional incapacity score' (FIS) was defined as the sum of the three categorical incapacity variables. The FIS (score 0-6) reflects the severity of the incapacity in different functional areas (mental, physical, and autonomy) and follows approximately a Poisson distribution. 3) 'Maximum work disability class (MWDC)'. This outcome measure consists of two values: a) 80%-100% work disability and b) less than 80% work disability.

**Table 1:** Client characteristics: available background variables and outcome measures for all clients in the analysis* (n = 83755)

Client characteristic	Class	
Gender		
(background)	Man(= 1)(ref)	44.4%
	Woman (= 2)	55.6%
Age		
(background)	15-24 years	2.6%
	25-34 years	19.3%
	35-44 years	36.1%
	45-54 years	32.4%
	≥ 55 years	9.6%
	Mean (sd) median age (years)	42.5 (9.1) 43.0
Statutory regime		
(background)	WAO (= 1) (ref)	26.8%
	HERBO (= 2)	47.5%
	WIA (= 3)	25.7%
Sector of Occupation		
(background)	Agriculture/Fishery/Foods (= 1)	4.4%
	Construction (= 2)	6.3%
	Industry (= 3)	9.1%
	Retail/Wholesale (= 4)	9.4%
	Transport (= 5)	3.3%
	Financial Services(= 6)	6.1%
	Employment Agencies (= 7)	5.5%
	Health and Care(= 8)	14.1%
	Education(= 9)	3.0%
	Other Governmental (= 10)	4.8%
	Other company/profession (= 11) (ref)	34.2%
Diagnosis		
(background)	Cardiovascular (= 1)(ref)	5.1%
	Musculoskeletal (= 2)	32.2%
	Mental (= 3)	34.3%
	Other(= 4)	28.5%
Objectification of Complaints		
(background)	Yes (= 0) (ref)	48.1%
	No (= 1)	51.9%
No Sustainable Capacities Left Extended		
(outcome measure)	No (= 0) (ref)	65.0%
	Yes (= 1)	35.0%
Maximum Work Disability Class		
(outcome measure)	< 80% (= 0) (ref)	57.2%
	80 - 100% (= 1)	42.8%

* Variables of which we present the mean, the standard deviation and the median are continuous and (approximately) normally distributed; the other variables are categorical; ref = reference category.

**Table 2:** Client characteristics: available background variables and outcome measures for part of the clients in the analysis* (n = 64190 - 64398)

Client characteristic	Class	
Standard Earnings per month		
(background)	€ 0 - € 917 (= 1)	10.1%
	€ 918 - € 1127 (= 2)	22.4%
	€ 1128 - € 1535 (= 3)	35.3%
	€ 1535 - € 2069 (= 4)	22.5%
	(€ 2070 - highest (= 5)	9.7%
	Mean (sd) median score (scale 1..5)	3.0 (1.1) 3.0
Standard Working Hours per week		
(background)	≤ 20 hours (= 1)	13.1%
	21 thru 32 hours (= 2)	21.5%
	32 thru 38 hours (= 3)	44.0%
	39 thru 40 hours (= 4)	16.6%
	> 40 hours (= 5)	4.8%
	Mean (sd) median score (scale = 1..5)	2.8 (1.0) 3.0
Education Level		
(background)	Grade 1(= 1)	4.4%
	Grade 2 (= 2)	23.1%
	Grade 3(= 3)	31.8%
	Grade 4 and 5 (= 4)	30.1%
	Grade 6 and 7(= 5)	10.6%
	Mean (sd) median score (scale 1..5)	3.2 (1.0) 3.0
Mental Incapacities		
(background)	Not or Slightly (= 0) (ref)	85.6%
	Moderate (= 1)	7.5%
	Rather strong or Strong (= 2)	6.8%
Physical Incapacities		
(background)	Not or Slightly (= 0) (ref)	84.6%
	Moderate (= 1)	5.6%
	Rather strong or Strong (= 2)	9.8%
Autonomous Incapacities		
(background)	Not or Slightly (= 0) (ref)	92.3%
	Moderate (= 1)	3.5%
	Rather strong or Strong (= 2)	4.2%
Functional Incapacity Score		
(outcome measure)	(Nearly) none incapacities (= 0)	69.9%
	Score (1)	11.1%
	Score (2)	13.1%
	Score (3)	2.8%
	Score (4)	2.7%
	Score (5)	0.2%
	Maximum severe incapacities (= 6)	0.1%
	Mean (sd) median score (scale 0..6)	0.6 (1.0) 0.0

* Variables of which we present the mean, the standard deviation and the median are continuous and (approximately) normally distributed; the other variables are categorical; ref = reference category. This part of the total file concerns the clients of whom the functional disability was assessed by an insurance physician using the List of Functional Abilities (LFA) and was administrated in the Claim Assessment and Monitoring System (CAMS).

### Client background variables

The client background variables are summarized in Table 1. The background variables available for all clients were: gender, ten-year age group, sector of occupation, diagnosis group and objectification of complaints. The actual age was missing for 85 clients. To use the actual age for all clients in the analysis, we then assigned these 85 clients to the midpoints of the ten-year age group to which they belonged. The diagnosis group variable was constructed on the basis of the primary diagnosis for each client. The binary variable 'objectification of complaints' was constructed on the basis of insights shared by the medical staff at the Dutch Employee Insurance Authority on complaints that are difficult to objectify in practice, i.e. medically unexplained symptoms or symptoms that do not have a clear medical cause (e.g. the chronic fatigue syndrome).

The other background variables - standard earnings, standard working hours, educational level, and the severity of the three distinguished incapacities (see Table 2) - were available for about 77% of the clients. The insurance physicians had completed a FAL for these clients. As was explained before with the construction of the outcome measure FIS, we formed three categorical incapacity variables from the items of the FAL: mental incapacities, physical incapacities and incapacity to function autonomously, each scored from null to two.

The labour expert added information to the database on the insured standard income of the client, the client's educational level and the number of hours previously worked by the client. We recoded this information into three approximately normally distributed variables.

### Insurance physician variables

Table 3 contains a summary of the survey-based insurance physician variables which are relevant to this study. As the method for measuring these variables was explained in detail elsewhere [9,10], we shall confine ourselves here to an indication of the meaning of the ASE variables.

**Table 3:** Characteristics of insurance physicians who gave informed consent: background variables and ASE-variables* (n = 199)

Background variables		
Gender	Man	58.8%
	Woman	41.2%
Age	30-45 years	23.6%
	46-50 years	27.7%
	51-55 years	25.0%
	≥ 56 years	23.7%
	Mean (sd) median age (years)	50.7 (7.0) 50.4
Career as Insurance Physician*	0-10 years	24.1%
	11-15 years	25.6%
	16-20 years	26.2%
	≥ 21 years	24.1%
	Mean (sd) median career (years)	16.0 (7.7) 16.5
Former Curative Physician	No (= 0)	84.4%
	Yes (= 1)	15.6%
Specialist in Insurance Medicine	No (= 0)	13.6%
	Yes (= 1)	86.4%
Standard working hours per week	< 25 hours (= 1)	18.1%
	25-32 hours (= 2)	23.1%
	> 32 hours (= 3)	58.8%
Most clients from WIA	No (= 0)	64.3%
	Yes (= 1)	35.7%
Most clients from WAO	No (= 0)	70.4%
	Yes (= 1)	29.6%
Most clients from WAJONG	No (= 0)	86.9.%
	Yes (= 1)	13.1%
Average number of assessments per week	1-5	23.1%
	6-15	29.7%
	16-22	25.6%
	23-28	21.6%
	Mean (sd) median assessments (number)	14.0 (8.4) 15.0

ASE variables**		

Attitude	Mean (sd) median	0.0 (1.0) 0.0
Social Norm	Mean (sd) median	0.0 (1.0) 0.0
self-Efficacy	Mean (sd) median	17.6 (3.2) 18.0
Barriers	Mean (sd) median	0.0 (1.0) 0.0
Knowledge	Mean (sd) median	0.0 (1.0) 0.0
Intention	Mean (sd) median	0.0 (1.0) 0.0
Behaviour: process	Mean (sd) median	0.0 (1.0) 0.0
Behaviour: assessment	Mean (sd) median	0.0 (1.0) 0.0

* Variables of which we present the mean, the standard deviation and the median are continuous and (approximately) normally distributed; the other variables are categorical.

** Factor scores, normalized resulting from measurement models (with Lisrel) for the (latent) ASE-variables, each loading on three or more additive scales and Homals-dimensions; see references [10,11]. Self-Efficacy is not normalized because this variable consists of one additive scale only, normally distributed.

The implications of a higher score for the eight ASE variables are explained below:

• Attitude: the insurance physician has a more positive attitude towards the profession of insurance physician, the quality of one's work, the professional staff, and the (Dutch) social insurance system;

• Social Norm: the insurance physician performed more autonomously, less influenced or uninfluenced by the social environment (colleagues, organization, society);

• Self-Efficacy: the insurance physician has more confidence in his/her own ability to control the interaction with the client during the assessment interview;

• Barriers: the insurance physician experiences more barriers arising from work pressure, emotional workload, less scope for decision-making, higher levels of burnout, poorer cooperation in the office, less incentives from management and relatively more 'difficult' clients;

• Knowledge: the insurance physician has sufficient information about the medical status of the client and about the rehabilitation efforts of the employer and the client;

• Intention: the insurance physician attaches more importance to: the promotion of recovery, resumption of work, self-reflection and re-integration, the relevance of capability, illness, disorders and handicaps in the assessment and, most of all, proper checks for the consistency of information on the daily activities and home situation of the client. To some extent this paints the professional attitude that one would expect from an insurance physician;

• Behaviour concerning the assessment process (Behaviour: process): the insurance physician has more confidence in his/her own vision of the job. A higher score suggests an insurance physician who is eager, takes control and is prepared to strike a compromise with the client;

• Behaviour concerning the assessment content (Behaviour: assessment): the insurance physician adheres more to rules and professional standards. A higher score is typical of an insurance physician who sticks to the guidelines, does not consider the specific situation of the client, thinks as he goes along, but still believes that he engages with the client.

### Analysis

We performed a non-response analysis for the insurance physicians and the clients. We compared relevant background variables of the insurance physicians in the study population with the available characteristics of all the insurance physicians at the Employee Insurance Authority. We also analysed the difference between the physicians whose survey results could and could not be linked with the database.

We compared the data on the clients in our analysis with the data of all the clients in the database across the same assessment periods for the three regimes. We used frequencies for all variables, crosstabs and Chi-squared statistics for categorical variables, and means and t-tests for continuous variables, using SPSS 15.0.

Using crosstabs and Chi-squares, we analysed the outcome measures univariately across insurance physicians and offices. We calculated the (arithmetical) mean, the standard deviation, the minimum and maximum and variation coefficient (standard deviation divided by the mean) across insurance physicians and offices by extracting the row percentages of the interesting categories in the categorical outcome measures from the crosstabs tables. For the univariate, descriptive analysis only, we divided the (approximately Poisson distributed) Functional Incapacity Scores (0 up to 6) into three parts: (nearly) no incapacity (Functional Incapacity Score 0), moderate incapacity scores (Functional Incapacity Scores 1 up to 3) and high incapacity scores (Functional Incapacity Scores 4 up to 6). We produced these descriptives with SPSS 15.0 (crosstabs tables and Chi-square statistics) to derive the row percentages and used MS Office Excel 2003 to calculate their central tendency and dispersion.

We performed multilevel analysis with MLwin version 2.02 [27] for the outcome measures, ranging from low to high, for client level, insurance physician level and office level.

We used logistic regression to analyse the two dichotomous outcome measures (NSCE, MWDC) and Poisson regression to analyse the outcome measure approximating a Poisson distribution (FIS). Because not all client background variables were available for all the outcome measures of clients, these outcome measures were analysed with different sets of client background variables. We analysed NSCE with Gender, Age, Sector of Occupation, Diagnosis, Objectification of Complaints and Statutory Regime as independent client background variables. The outcome measure (FIS) for the group of clients with a FAL (about 77%) was analysed with the fore-mentioned client background variables plus Standard Earnings, Standard Working Hours and Education Level. The MWDC outcome measure was analysed for the same 77% of clients and with the same set of independent client background variables plus Mental Incapacities, Physical Incapacities and Autonomous Incapacities.

We estimated all the multivariate models in MLwin with Restricted Maximum Likelihood (RIGLS), the 'naive' models with first-order maximized quasi-likelihood (MQL) and the multilevel models with second-order penalized quasi-likelihood (PQL). The significance of coefficients was determined with Wald statistics. We chose P ≤ 0.10 as the level of significance because we did not want to miss interesting associations.

### Model specifications

The multilevel analysis for each outcome measure consisted of four steps [28]. Firstly, we performed a "naïve" analysis, i.e. an analysis without random coefficients, with all the available client background variables, including an intercept, regardless of whether or not they were significant. Secondly, we added random coefficients for the intercept and for the client background variables one by one, first for the physician level and then for the office level, in that order; significant random coefficients remained in the model. Thirdly, we added the variables of the insurance physicians one by one, beginning with the background variables of the insurance physicians and then the ASE variables; significant coefficients remained in the model. We also added two physician-client interaction terms for gender and age. Fourthly, we added random coefficients at office level for the insurance physician variables one by one; significant random coefficients stayed in the model.

We expected Attitude, Intention and Behaviour: assessment to relate negatively to the outcome measures, and Behaviour: process, which showed a weak negative relationship with Behaviour: assessment [10], to relate positively to them. We had no clear expectations of the tendency of the associations of the other ASE variables.

### Intra-Class Correlations

To determine the associations of the outcome measures at insurance physician level and office level, we calculated intra-class correlations (ICCs), which are commonly used in research to determine the extent of systematic variations between elements at a higher level [29-31]. In this study an ICC indicates the association between an outcome measure for clients who have been assessed by the same insurance physician (IP level) and by different insurance physicians within the same office (office level).

An ICC is defined as the variance in the outcome measures (at client level) between physicians (or offices) divided by the total variance (the sum of the 'between' and the 'within' variance). The greater the 'between' variance is, the smaller the 'within' variance and the greater the ICC. An ICC that edges towards one, means that the 'between' variance is almost equal to the total variance. In our study this would indicate that the assessment outcomes are determined at insurance physician level (or office level). Conversely, an ICC that edges towards zero means that the 'within' variance is almost equal to the total variance. In our study this would indicate no association at insurance physician level (or office level) with the assessment outcomes of the clients. Additional file 1 contains a more detailed explanation of how we calculated the ICCs for logistic regression and Poisson regression.

To gain an indication of the contribution of the client background variables to the total explained variance of the outcome measures we calculated the difference between the total explained variance on the one hand and the ICCs for IP level and office level on the other for the various models.

Usually, in 'real life' cross-sectional studies among physicians, the ICC is no higher than 0.20 [28]. This is observable in an ICC database in the UK [32], containing empirical estimates of ICCs calculated from a number of datasets from primary and secondary healthcare implementation studies [33]. In order to evaluate the ICCs for insurance physicians in comparison with physicians in general, we consider ICCs as low if ≤ 0.10, as moderate if 0.10 < ICC ≤ 0.20, and as high if > 0.20.

## Results

### Non-response

Because the necessary data of the target population (N = approximately 450) to do a full non-response analysis were not available, Steenbeek et al. [9] checked whether the group of participants (N = 231, approximately 51% of the target group) was representative of the total population of insurance physicians working at the Employee Insurance Authority (N = approximately 900, including staff-members and physicians not performing disability assessments) in terms of age, gender, and working hours per week. They concluded that the group of 231 physicians was representative of this total population of insurance physicians at the Employee Insurance Authority in these terms. Furthermore, the group of 199 insurance physicians who gave consent to link survey answers to their client assessment data (approximately 44% of the target group) resembled this total population as well in terms of age (51 versus 49 years in total population), gender (41% versus 42% females in total population), and working hours per week (31 versus 32 hours in total population).

This raises the question whether the 32 insurance physicians who did not give us informed consent to link their survey data with the database (including one physician for whom the link failed) differed from the group of 199 insurance physicians. There was a significant relationship between these groups in terms of: a) most of the clients were assessed for the WAO (overrepresented in the analysed group of 199 physicians; P = 0.005); b) most of the clients came from the temporary work sector (underrepresented in the analysed group; P = 0.065); and c) most of the clients came from public sectors other than healthcare and education (overrepresented in the analysed group; P = 0.074). As far as age and the eight ASE variables were concerned, the analysed group of 199 physicians experienced fewer Barriers (P = 0.003), had higher self-Efficacy (P = 0.027) and scored higher for sufficient Knowledge (P = 0.035) than the group of 32 physicians. The variances for the continuous variables did not differ between the two groups.

In our analysis of the non-response clients we compared the data of the clients of the 199 IPs with the data of all the clients over the same period. Only minor differences emerged between the variables of the 83,755 clients in our analysis as shown in Table 1, compared with all 355,757 clients in the database. There were more WIA assessments (+3.6%), fewer WAO assessments (-3.1%) and more clients in the 45-54 age group (+3.1%) in our analysed client group than in the total group.

### Descriptives of the outcome measures

The outcome measures for clients are listed in Table 1 and Table 2. We analysed the central tendency and the dispersion at physician level and office level (see Table 4). We were especially interested in the variation coefficients, i.e. the standard deviation divided by the mean, because they indicate the degree of uncorrected variation in the outcome measures for the two levels.

**Table 4:** Central tendency and dispersion of outcome measures for clients across insurance physicians (n = 199) and across offices (n = 27)*

Outcome measures	NSCE	FIS0	FIS123	FIS456	MWDC
**Across insurance physicians**					
Mean	35.1	69.6	27.3	4.1	42.9
median	34.1	72.0	25.9	2.0	42.9
minimum	8.6	22.4	4.8	0.5	10.5
maximum	83.3	95.2	61.2	19.3	86.1
standard deviation	11.9	11.0	9.0	3.0	9.8
variation coefficient	0.34	0.16	0.33	0.75	0.23

**Across offices**					
Mean	34.7	68.6	28.1	3.3	43.9
median	33.3	68.5	28.3	3.2	42.7
minimum	18.2	44.6	20.7	0.9	35.1
maximum	49.9	78.3	47.3	8.8	53.0
standard deviation	8.3	6.7	5.1	2.0	4.4
variation coefficient	0.24	0.10	0.18	0.60	0.10

* Row percentage across insurance physicians and across offices respectively, by categories (yes) of outcome measures. Number of clients: n = 83755 clients for NCSE and MDWC; n = 64190 clients for FIS. NSCE = no sustainable (long-term) functional capacities anymore, or restrictions in working hours up to 30 hours (category = yes); FIS0 = functional incapacity score (score 0 = yes); FIS123 = functional incapacity score (score 1 up to 3 = yes); FIS456 = functional incapacity score (score 4 up to 6 = yes); MWDC = maximum work disability class 80%-100% (category = yes).

At physician level and office level (see Table 4) the highest variation coefficient was for the category of clients with high incapacity scores (FIS456). The variation coefficients at office level are of course lower than at physician level.

### Multilevel analyses

Table 5, Table 6 and Table 7 show the models of the MLwin analyses for each of the three outcome measures: NSCE, FIS and MWDC, successively. The tables show the coefficients of all available client background variables in the model. However, only variables that reach significance level were used (P ≤ 0.10) for the insurance physician. Here we concentrate on the results at insurance physician level.

**Table 5:** Results Multilevel Analysis for No Sustainable Capacity or Restriction in Working Hours (NSCE, n = 83755)

	Fixed			Random	
				IP	Office
	
	OR	95% CI	p	p	p
***Client (CL)***					
Constant	0.647	0.531-0.789	****	****	***
Gender_CL (female)	1.340	1.276-1.408	****	****	
Age_CL/10	1.036	1.012-1.061	***	****	
Statutory regime HERBO (yes)	1.009	0.927-1.098	NS	****	
Statutory regime WIA (yes)	1.169	1.070-1.277	****	****	
Agriculture/Fishery/Foods (yes)	1.035	0.931-1.150	NS		
Construction (yes)	0.834	0.739-0.942	***		
Industry (yes)	0.965	0.888-1.047	NS		
Retail/Wholesale (yes)	1.197	1.105-1.297	****		
Transport (yes)	0.842	0.753-0.942	***		
Financial Services (yes)	1.139	1.045-1.241	***		
Employment Agencies (yes)	1.215	1.108-1.333	****		
Health and Care (yes)	1.121	1.030-1.219	***		
Education (yes)	1.154	1.008-1.321	**		
Other Governmental (yes)	1.287	1.157-1.430	****		
Musculoskeletal diseases (yes)	0.264	0.243-0.288	****	****	
Mental diseases (yes)	1.632	1.480-1.800	****	****	
Other diseases (yes)	1.276	1.189-1.370	****		
Objectification of Complaints (yes)	0.342	0.318-0.368	****	****	

***Insurance Physician (IP)***					
Attitude	0.950	0.896-1.008	*	NA	

CL = client; IP = insurance physician level; Office = office level; OR = Odds Ratio; CI = confidence interval; p = two-tailed probability; NA = not applicable; NS = not significant.

* = 0.05 < p ≤ 0.10; ** = 0.01 < p ≤ 0.05; *** = 0.001 < p ≤ 0.01; **** = p ≤ 0.001

**Table 6:** Results Multilevel Analysis for Functional Incapacity Score (FIS, n = 64190)

	Fixed			Random	
				IP	Office
	
	OR	95% CI	p	p	p
***Client (CL)***					
Constant	0.338	0.274-0.417	****	****	
Gender_CL (female)	1.046	1.008-1.086	**	****	
Age_CL/10	1.156	1.130-1.183	****	****	
Statutory regime HERBO (yes)	1.968	1.809-2.141	****	****	
Statutory regime WIA (yes)	1.511	1.381-1.654	****	****	
Agriculture/Fishery/Foods (yes)	0.998	0.935-1.065	NS		
Construction (yes)	0.862	0.797-0.933	****		
Industry (yes)	0.893	0.849-0.940	****		
Retail/Wholesale (yes)	0.941	0.892-0.992	**		
Transport (yes)	0.810	0.750-0.874	****		
Financial Services (yes)	0.924	0.871-0.980	***		
Employment Agencies (yes)	0.900	0.851-0.953	****		
Health and Care (yes)	0.969	0.914-1.028	NS		
Education (yes)	1.068	0.965-1.183	NS		
Other Governmental (yes)	1.135	1.062-1.214	****		
Musculoskeletal diseases (yes)	0.697	0.644-0.754	****	****	
Mental diseases (yes)	1.772	1.629-1.928	****	****	
Other diseases (yes)	0.899	0.832-0.970	***	****	
Objectification of Complaints (yes)	0.560	0.530-0.591	****	****	
Standard Earnings	0.880	0.863-0.897	****	****	
Standard Working Hours	0.978	0.961-0.996	**		
Education level	0.956	0.941-0.971	****	****	

***Insurance Physician (IP)***					
Years Career as IP	1.009	1.003-1.015	***	NA	

CL = client; IP = insurance physician level; Office = office level; OR = Odds Ratio; CI = confidence interval; p = two-tailed probability; NA = not applicable; NS = not significant.

* = 0.05 < p ≤ 0.10; ** = 0.01 < p ≤ 0.05; *** = 0.001 < p ≤ 0.01; **** = p ≤ 0.001

**Table 7:** Results Multilevel Analysis for Work Disability Class 80%-100% (MWDC, n = 64190)

	Fixed			Random	
				IP	Office
	
	OR	95% CI	p	p	p
***Client (CL)***					
Constant	2.832	1.004-7.987	**	***	
Gender_CL (female)	0.918	0.870-0.967	***		
Age_CL/10	0.978	0.801-1.196	NS	**	
Legal regime HERBO (yes)	0.945	0.858-1.040	NS	****	
Statutory regime HERBO (yes)	0.610	0.540-0.688	****	****	
Statutory regime WIA (yes)	1.134	0.995-1.293	*		
Construction (yes)	0.845	0.732-0.974	**		
Industry (yes)	0.882	0.795-0.979	**		
Retail/Wholesale (yes)	0.967	0.875-1.068	NS		
Transport (yes)	0.772	0.670-0.889	****		
Financial Services (yes)	0.919	0.817-1.034	NS		
Employment Agencies (yes)	1.084	0.968-1.215	NS		
Health and Care (yes)	0.920	0.826-1.025	NS		
Education (yes)	0.854	0.701-1.041	NS		
Other Governmental (yes)	0.805	0.698-0.929	***		
Musculoskeletal diseases (yes)	0.594	0.537-0.656	****	**	
Mental diseases (yes)	1.134	1.008-1.276	**	***	
Other diseases (yes)	1.122	1.017-1.237	**		
Objectification of Complaints (yes)	0.615	0.577-0.656	****	***	
Mental Incapacities moderate (yes)	5.496	4.944-6.109	****	****	
Mental Incapacities strong (yes)	13.929	12.144-15.978	****	****	
Physical Incapacities moderate (yes)	5.960	5.446-6.522	****	**	
Physical Incapacities strong (yes)	21.520	19.898-23.275	****		
Autonomous Incapacities moderate (yes)	2.472	2.181-2.802	****		
Autonomous Incapacities strong (yes)	4.730	4.173-5.363	****	**	
Standard Earnings	0.828	0.805-0.851	****		
Standard Working Hours	0.959	0.937-0.982	****		
Education level	0.777	0.753-0.802	****	****	

***Insurance Physician (IP)***					
Age_IP/10	0.819	0.673-0.996	**	NA	
Age_IP*Age_CL/100	1.039	0.999-1.080	*	NA	
Former Curative Physician (yes)	1.150	1.003-1.319	**	NA	
Self-efficacy	0.988	0.975-1.002	*	NA	
Knowledge	1.057	1.006-1.110	**	NA	
Behaviour: assessment	1.070	1.017-1.126	***	NA	

CL = client; IP = insurance physician level; Office = office level; OR = Odds Ratio; CI = confidence interval; p = two-tailed probability; NA = not applicable; NS = not significant.

* = 0.05 < p ≤ 0.10; ** = 0.01 < p ≤ 0.05; *** = 0.001 < p ≤ 0.01; **** = p ≤ 0.001

At IP level, most of the client background variables, except sector of occupation, showed a strong significant association (P ≤ 0.01) with the outcome measures. No association was found at IP level for gender (for MWDC), other diseases (for NSCE and FIS), strong physical incapacities, moderate autonomous incapacities and standard earnings (for MWDC) and standard working hours (for FIS and MWDC). At office level, only one random coefficient for the constant turned out to be weakly significant (P ≤ 0.10; for NCSE), indicating that there was a small systematic difference for this outcome measure between offices. None of the fixed coefficients for the independent insurance physician variables was associated with office level, indicating that there were no systematic differences in the outcome measures between offices, because of these insurance physician variables.

We found only a few, weak associations between the outcome measures and the independent insurance physician variables (see Table 5, Table 6 and Table 7). In the case of the background physician variables we found a weak positive interaction effect for MWDC between the age of the physician and the age of the client (see Table 7). Hence, when both the insurance physician and the client belonged to an older age group, more MWDC assessments could be expected. Previous employment as a curative physician was also positively associated with more MWDC assessments. A longer career as insurance physician was associated with a higher Functional Incapacity Score (FIS) (see Table 6).

No associations were found between the three outcome measures and the ASE insurance physician variables: Social Norm, Barriers, Intentions and Behaviour: process (see Table 5, Table 6 and Table 7). A more positive Attitude, i.e. towards the profession, the quality of the work and the social insurance system, was weakly associated with fewer NSCE assessments (see Table 5). More self-Efficacy was weakly associated with less MWDC assessments (see Table 7). A higher score for Knowledge, i.e. possessing sufficient information about the client, and a higher score for Behaviour: assessment, i.e. more adherence to rules and professional standards, was also associated with more MWDC assessments (see Table 7).

We calculated Intra Class Correlations (ICCs) for the association between each of the three outcome measures on the one hand and insurance physician level and office level on the other. These ICCs are shown in Table 8 together with the explained variance (R^2^). The ICCs ranged between 6.5% and 11.8%. The highest ICC at IP level was for Functional Incapacity Score (FIS). The contribution of the insurance physician background variables and the ASE variables to the ICCs at physician level was very small, around 1% (not in Table 8).

**Table 8:** Intra Class Correlations and explained variances (in percentages), calculated on basis of prediction

Dependent variables	NSCE (n = 83755)	FIS (n = 64190)	MWDC (n = 64190)
ICC IP-level	7.1%	11.8%	6.5%
ICC Office-level	1.6%	0.0%	0.0%
R^2 ^total model	19.1%	18.5%	37.2%
Contribution in R^2 ^by client variables*	10.4%	6.7%	30.7%

NSCE = no sustainable (long-term) functional capacities anymore, or restrictions in working hours up to 30 hours (binominal); FIS = functional incapacity score (scale); MWDC = maximum work disability classes 80%-100% (binominal); ICC = Intra Class Correlation; IP = Insurance Physician; R^2 ^= explained variances. The ICCs for NSCE and MWDC were estimated with logistic regression; that of FIS with Poisson regression.

*calculated as the difference between explained variances and ICCs (IP-level and office level).

At office level only an ICC for No Sustainable Capacities Extended (NSCE), though rather small, was to some extent meaningful. For the other two outcome measures, Functional Incapacity Score (FIS) and Maximum Work Disability Class (MWDC) the ICCs at office level were zero. The explained variance (R^2^) was highest (37%) for Maximum Work Disability Class (MWDC). The contribution of the client background variables to the explained variance of this outcome measure was nearly 31%. The three client background (incapacity) variables in the model were strongly associated with MWDC: Without these variables the explained variance of MWDC worked out at around 14% with a contribution of around 8% by the client background variables (not in Table 8).

## Discussion

### Answering the research questions

The four research questions of our study can be answered successively. Firstly, the variation in the outcome measures of disability assessments at IP level was univariately highest for clients with a high Functional Incapacity Score (FIS456). The same result was found for the univariate variation at office level.

Secondly, the strength of association between the client background variables and the outcome measures, i.e. the explained variance, was 10% in the case of No Sustainable Capacity Extended (NSCE) and 6% in the case of Functional Disability Score (FIS). This contribution was highest - 31% - for Maximum Work Disability Class (MWDC). The three client background incapacity variables had very strong positive associations with Maximum Work Disability Class (MWDC).

Thirdly, the insurance physician background variables that were associated with the outcome measures are: the IP's age interacting with the client's age (weakly), previous employment as a curative physician and length of career as an insurance physician. Only four ASE variables were associated with the outcome measures: a positive Attitude was associated with lower scores for No Sustainable Capacities Extended (NSCE), higher self-Efficacy was weakly associated with fewer MWDC assessments, while a higher score for Knowledge, i.e. possessing sufficient information about the client, and a higher score for Behaviour: assessment, i.e. more adherence to rules and professional standards, was associated with more MWDC assessments. The contribution of the insurance physician background variables and the ASE variables to the ICCs at IP level were very small, at around 1%.

At last, on the basis of the previously mentioned ICC evaluation criteria, the following conclusions may be drawn: none of the ICCs for the outcome measures at insurance physician level can be qualified as high; only the ICC for Functional Incapacity Score (FIS) is moderate, i.e. around 12%; the ICCs at office level are very low, only the ICC for No Sustainable Capacities Extended (NSCE) is meaningful at 1.6%.

### Strengths and weaknesses of this study

One of the strengths of this study is that the insurance physicians (N = 199) are considered representative of all the insurance physicians at the Employee Insurance Authority. It should still be noted, however, that some differences emerged between the opinions of this group and those of the insurance physicians (N = 32) with no link to the client's database. The variances in the continuous variables did not, however, differ between these groups. Furthermore, only small differences emerged between the clients (N = 83,755) in our study and the total number of clients (N = 355,757). It is therefore unlikely that these differences will have a substantial effect on the estimation results of the multilevel analyses. Another strength is that the data covered a large number of clients and could be regarded as reliable since they came from an official database of the Employee Insurance Authority. The outcome measures that we used are relevant to the work of the insurance physicians at the Employee Insurance Authority and have deep relevance for Dutch society given the public expenditure on disability benefits. A third strength lies in the linkage between the client's data and the survey data of insurance physicians, and the reliability and validity of the ASE variables, which were constructed on the basis of measurement models and a structural equation [9,10]. Finally, as we used multilevel analysis (three levels), we were able to analyse the associations with insurance physician level, while correcting for associations between the outcome measures and the other two levels (client and office).

There are also some weaknesses in our study. The main weakness is the cross-sectional design, which prevents us from drawing conclusions about causal relationships. Secondly, we do not know whether the scores for the ASE variables based on the survey data of May 2008 are stable over time and valid for the preceding years. Another possible weakness may lie in the fact that the eight ASE variables are factor scores at such a highly condensed level, i.e. second-order factor scores of 48 additive scales and dimension variables resulting from first-order factor analysis and homogeneous analysis, that the relationship with the outcome measures might become very weak. We do not, however, believe this to be the case, as additional multilevel analyses with models, including the original 48 scales and dimension variables instead of the eight ASE variables, delivered similar results to those of the eight ASE variables. Another weakness was that we did not have client data on co-morbidity and the severity of the disorders because these were either poorly registered or not registered at all in the database by the insurance physicians. Such data could have influenced the results and accounted for lower explained variances, especially in the models which did not include the three incapacity variables.

A final weakness lies in the fact that we assigned each insurance physician to the office where he/she assessed the most clients, which might cause an overestimation of the ICC on the office level. However, because of the very low resulting ICCs for the office level, in our opinion this overestimation is negligible.

### Discussion of results

#### Insurance physician variables

Some associations were found between the background variables and ASE variables of the insurance physicians and the outcome measures (see Table 5, Table 6 and Table 7). However, these variables had only a minor impact on the explained variance of the outcome measures. Adding these insurance physician variables to the model has an effect only on the strength of the fixed coefficient of the constant and almost no effect on the fixed coefficients of the client variables or the random coefficients (variances) at IP level. Remarkably, there are no repeated associated variables at the insurance physician level for each of the outcome measures.

The associations between the insurance physician variables and the outcome measures give pause for thought if we interpret them as causal relationships. A longer career as an insurance physician leads to a higher Functional Incapacity Score (FIS, see Table 6). Previous employment as a curative physician leads to more assessments in Maximum Work Disability Class 80%-100% (MWDC; see Table 7). An older physician who assesses older clients produces more MWDC assessments (see Table 7). It is unclear whether these associations should be interpreted as causal or selection effects. However, in our opinion, the associations between the ASE variables of the insurance physicians and the outcome measures can only be meaningfully interpreted as causal relationships.

Firstly, a positive attitude towards one's work as insurance physician, towards the quality of the work and towards the social insurance system leads, according to our expectations, to fewer overall NSCE assessments (No Sustainable Capacities Extended; see Table 5). Perhaps these insurance physicians take their profession, the quality of their work and the regulations (including the evidence based notion that work is good for somebody's health) more seriously and thus assess fewer clients as NSCE. Secondly, more self-Efficacy leads to fewer MWDC assessments (Maximum Work Disability Class 80%-100%; see Table 7). It is conceivable that, in this case, the insurance physicians are so confident that they can resist clients who claim to be fully disabled. Thirdly, adherence to rules (Behaviour: assessment) leads more often to MWDC assessments (see Table 7). More information about the medical status of the client and about the rehabilitation efforts of the employer and the client (Knowledge), which relates positively to rule adherence (Behaviour: assessment) in the structural equation model [10], is also more likely to lead to MWDC assessments (see Table 7). More information (e.g. from the occupational health physician or from the reintegration report) may be associated with more incapacitated clients. Adherence to rules, in the case of these clients, may then imply a higher chance of an MWDC assessment. Our findings seem in accordance with those of Spanjer [14], who found in his study that *additional *information provided by the patient on participation and on activity limitations, led to significantly more scores in the assessment on work limitation items compared with information from the medical history-taking alone. More research is needed to elucidate the role of extra information in our study.

The finding that ASE variables appear to have only weak effects on the outcome measures might be explained by the assumption that self-reported data on attitudes, intentions and behaviour have weaker or weak associations with registered outcome data because of the different nature of both [34,35]. In our opinion, this explanation does not apply here because the background insurance physician variables are also weakly associated with the outcome measures. We are therefore inclined to consider the weak relationships between the insurance physician variables and the outcome measures as 'real' and 'true'; in other words, the individual characteristics and opinions of insurance physicians have very little impact on the outcome of disability assessments in the social insurance context in the Netherlands.

#### ICCs and explained variance

The ICC at insurance physician level is highest for Functional Incapacity Score (FIS), but may still be qualified as moderate. This ICC may be relatively high because of the absence of guidelines or protocols on how to quantify restrictions on working hours and the severity of incapacities. This absence, in itself, is hardly surprising, given that there is little or no evidence that certain diseases lead to certain disabilities with specific degrees of severity. Dutch social insurance physicians even have difficulty reaching consensus on the level of disability arising from a major depression [36]. Conversely, for the other two outcome measures fairly extensive standards are available [37] for some time now. Giving our results, these standards do probably lead to lower ICCs at IP level.

The ICCs at office level are either low or negligible, which means that regional managers did not influence the assessment results in any other way than they are instructed to do in centralized directives from the Board of the Dutch Employee Insurance Authority. Our results show that the apparent, univariate differences between offices can to some extent be explained by differences at client and insurance physician level.

Even though the ICCs at IP level are evaluated as low to moderate, they still form the largest part of the total explained variance for most of the outcome measures and thus suggest that insurance physician level is the prime factor in the apparent differences between insurance physicians. We refute this argument because it implies that if the explained variances were increased by, for example, implementing health variables in the model for these outcome measures, the ICCs at IP level would be higher as well. The results for Maximum Work Disability Class compared with those of Functional Incapacity Score show that this is not the case. It could also be argued that the ICCs at IP level are low because of the very large differences in the assessment results for the same kind of clients by the same insurance physicians. Again, we refute this theory: the results showed a very strong and consistent relationship between the three incapacity variables and Maximum Work Disability Class. This argument is also rendered invalid by research on the reliability of the FAL by Spanjer [12-14,16], who found average absolute agreements of 74% to 84% among insurance physicians.

In our study we found an average ICC of 12% at insurance physician level for Functional Incapacity Score: systematic variations between the overall incapacity assessments of clients with all types of disorders, which have to be attributed to differences between insurance physicians. This ICC of 12% reflects a systematic difference in a minority of the assessment outcomes with the FAL. This is in accordance with the absolute agreement found in the referred studies by Spanjer for the majority of the assessments.

### Practical relevance

What is the relevance of our findings on systematic inter-doctor variations? Because the background variables and the ASE variables of the insurance physicians account for only about 1.0%-point of the ICC of 12% for FIS, the systematic variations in assessments with the FAL must be due to other factors related to insurance physicians. One plausible explanation is that different insurance physicians have different work routines, i.e. the way insurance physicians routinely conduct their (physical and psychological) examinations of clients' health condition, which may result in different outcomes. Another plausible explanation is that insurance physicians may have different visions, consciously or unconsciously, of the workload of a 'normal' employee, which may result in different assessments of the incapacities and limitations. Or, the IPs may have different insights because of differences in the amount and accuracy of actual information from the client and employer. This includes information about the clients' social circumstances (e.g. caring responsibilities) and how the IP values this information in his assessment. IPs may differ in their expectations of what an employee should be able to cope with at work.

If these explanations are valid, the systematic variations may be narrowed down by well-developed and comprehensively implemented guidelines from the Employee Insurance Authority on how to assess the severity of disabilities [38]. At present, the professional society of insurance physicians in the Netherlands is developing protocols in the form of well-documented disability assessment cases, supported by consensus within the profession, which offer general assessment guidelines for similar cases. The trend towards more guidelines and case protocols picks up on Eisenberg's findings (1985; 2002), which suggest that less uncertainty (i.e. in our case, less uncertainty about how to assess the severity of disabilities) may lower the degree of variation. We believe that these proposals may be relevant for disability assessments in other countries as well. Further research is needed whether or not implementation and use of guidelines may lower the variation in assessment outcomes of functional capacity. If this proves to be valid in the Netherlands, the next step may be international research on this topic.

## Conclusions

The individual characteristics and opinions of insurance physicians have very little influence on the outcome of disability assessments in the Netherlands. That said, our study still revealed systematic variations, which can be qualified as 'normal', i.e. equal to those between physicians in primary and secondary care. Our findings provided no indication of the reasons for these variations. They may be related to different work routines or to different views on the workload of a 'normal' employee. If so, they could be reduced by well-developed and comprehensively implemented guidelines, particularly on the assessment of working hour restrictions and the severity of the disability. Further research is needed to determine whether this is a valid proposal.

## Competing interests

The authors declare that they have no competing interests.

## Authors' contributions

AJMS, HM and RS designed the study. AJMS performed the statistical analysis and wrote the manuscript and RS revised the manuscript. HM, JRA, HK and JB commented on the manuscript. RS, HM, HK and JB coordinated the data collection. HK and HM coordinated the procedures for anonymizing and linking the data. All authors read and approved the final manuscript.

## Pre-publication history

The pre-publication history for this paper can be accessed here:

http://www.biomedcentral.com/1471-2458/11/864/prepub

## Supplementary Material

Additional file 1 **Calculating Intra Class Correlations (ICCs)**. Explanation how the ICCs were calculated. Click here for file
