# Supplementary material for: Inter-doctor variations in the assessment of functional incapacities by insurance physicians
Source: BMC Public Health. 2011 Nov 14;11:864. doi: 10.1186/1471-2458-11-864 (PMC3276607; doi:10.1186/1471-2458-11-864)
Supplement: Additional file 1 — Calculating Intra Class Correlations (ICCs). Explanation how the ICCs were calculated. [file 1471-2458-11-864-S1.DOC]

**Additional file: Calculating Intra Class Correlations (ICCs)**

In linear regression models the ICC for a higher level (in our study: insurance physician and office level) can be calculated by using the difference between the error variance of the same models with and without random coefficients. Unfortunately, no total variance is given (in MLwin) for logistic and Poisson regression models.

There is no commonly accepted method for calculating ICCs in logistic and Poisson regression models [28]. One option is to use the estimated variance coefficient of the random intercept for a level, in which case the formula is: the between-error variance (σ^2^) divided by the sum of the between-error variance (σ^2^) and the intra-cluster variance, i.e. a third of the squared pi (π^2^/3), i.e. σ^2^/[σ^2^ + (π^2^/3)] [28, Heo M, Leon AC: Performance of a Mixed Effects Logistic Regression Model for Binary Outcomes With Unequal Cluster Size. Journal of Biopharmaceutical Statistics 2005, 15:513 - 526]. The between-error variance for a level is estimated with the variance coefficient of the random intercept for that level. For example, the between-error variance for insurance physician level is estimated in a model with a random intercept for IP level alone and with only fixed coefficients for the client background variables; hence, no IP variables. The between-error variance for office level is then estimated with the same model, but with a random intercept both for IP and office level.

As this calculation method is difficult to interpret when determining an ICC for a logistic and Poisson regression model [28], we chose a method that we considered more appropriate for the goal of this study, one that would take explicit account of the associations between the client variables and IP and office level. We therefore calculated the individual outcome measures of an estimated model using the prediction option in MLwin. First, we calculated the predicted outcome for the full model with random coefficients (intercept and/or slopes) for both office level and IP level, then the predicted outcome for an intermediate model with only random coefficients for IP level, and finally, for the model with only fixed coefficients for intercept and client background variables. All these predictions were made under *ceteris paribus* conditions, starting from the full model.

The predicted outcome measures took the form of logit(y_e_) for the logistic regression models and the form of ln(y_e_) for the Poisson regression models. We then transformed the predicted outcome measure y_e_ =1/(1+e^-z^) for the logistic model with z=logit(y_e_), and y_e_=e^z^ for the Poisson model with z= ln(y_e_) [27]. By calculating the squared correlation between the observed outcome measure y and the predicted outcome measure y_e_, we obtained the explained variance (R^2^) of each model. Hence, in the case of two models, e.g. model A with random coefficients for insurance physicians and model B with fixed coefficients, the ‘error variance’ is σ^2^_ea_=(1-R_a_^2^) and σ^2^_eb_=(1-R_b_^2^), with σ^2^_eb_ ≥ σ^2^_ea_ . We calculated the ICC for the level in question as (σ^2^_eb_- σ^2^_ea_)/ σ^2^_eb_. Here, the ICC for office level was calculated as the difference in the error variances between the full model, including random coefficients for office level and IP level, and the model with only random coefficients for IP level. The ICC for IP level was calculated as the difference in the error variances between the model with random coefficients for IP level and the model with only fixed coefficients for the client background variables. This procedure enabled us to take account of the possible influence of IP variables on the ICC at IP level.
